# Returning to work after a sickness absence due to cancer: a cohort study of salaried workers in Catalonia (Spain)

**DOI:** 10.1038/s41598-021-03368-8

**Published:** 2021-12-14

**Authors:** Amaya Ayala-Garcia, Laura Serra, Julio C. Hernando-Rodriguez, Fernando G. Benavides

**Affiliations:** 1 Center for Research in Occupational Health (CiSAL), Universitat Pompeu Fabra, Barcelona, Spain; 2 CIBER of Epidemiology and Public Health (CIBERESP), Madrid, Spain; 3 IMIM – Parc Salut Mar, Barcelona, Spain; 4 Research Group On Statistics, Econometrics and Health (GRECS), University of Girona, Girona, Spain

**Keywords:** Cancer epidemiology, Public health, Occupational health

## Abstract

Cancer incidence and survival rates have increased in the last decades and as a result, the number of working age people diagnosed with cancer who return to work. In this study the probability of accumulating days of employment and employment participation trajectories (EPTs) in a sample of salaried workers in Catalonia (Spain) who had a sickness absence (SA) due to cancer were compared to salaried workers with SA due to other diagnoses or without SA. Each individual with SA due to cancer between 2012 and 2015 was matched by age, sex, and onset of time at risk to a worker with SA due to other diagnoses and another worker without SA. Accumulated days of employment were measured, and negative binomial models were applied to assess differences between comparison groups. Latent class models were applied to identify EPTs and multinomial regression models to analyse the probability of belonging to one EPT of each group. Men and women without SA or with SA due to other diagnoses had at least a 9% higher probability of continuing in employment compared to workers who had a SA due to cancer, especially among men without SA (adjusted IRR 1.27, 95% CI 1.06‒1.53). Men without SA had the highest probability of having high stable EPT compared to workers who had a SA due to cancer (adjusted RRR 3.21, 95% CI 1.87‒5.50). Even though workers with SA due to cancer continue working afterwards, they do it less often than matched controls and with a less stable employment trajectory. Health and social protection systems should guaranty cancer survivors the opportunity to continue voluntary participation in the labour market.

## Introduction

The effect of cancer on paid work is a growing problem that needs adequate collaborative responses between health and social protection systems. Cancer is becoming a highly prevalent chronic disease with a 28% increase in its global incidence in the past few decades^1–3^. The survival rate is also increasing 3% per year^4, 5^, but cancer survivors suffer from long-lasting symptoms after the acute stage due to the disease and its treatment. In 2017, cancer caused an estimated 3,204,000 disability-adjusted life-years (DALYs)^6, 7^, impacting all dimensions of survivors’ life including their future working life.

Almost half of individuals diagnosed with cancer are of working age^8^. According to IARC estimations, the number of new cases among individuals aged 15–69 years worldwide was 221.6 per 100,000 in 2020^9^. Although cancer is more likely to appear in older populations, the risk increases from 45 years of age^10^. Therefore, the steady increase in working age individuals diagnosed with cancer are expected to increase the number of people returning to work after treatment. Especially considering the delay in legal retirement age and the increasing tendency of workers to work beyond it^11^, although for workers with cancer after or close to legal retirement age , ending their working life is more likely than for cancer free workers^12^.

Return to work (RTW) could be beneficial for survivors´ health due to an increased sense of purpose, higher self‐confidence, and stronger sense of social belonging associated with employment^13^. RTW after a cancer diagnosis and treatment is considered a good prognostic sign. A successful RTW process is influenced by disease and treatment-related factors^14–16^, sociodemographic variables (age, sex, education level, marital status), and work and employment conditions, such as size and ownership of the organisation, physical and emotional job demands, working hours, type of job, attitudes of colleagues, type of contract, sick leave duration, and previous periods of unemployment^17–19^. Most cancer survivors attempt to RTW after treatment^20^, and studies have shown a steady increase in RTW as time after diagnosis increases^21^.

There is a growing consensus that work after cancer-survivorship research should address long-term work-related factors to understand the impact of cancer on the whole labour trajectory^22^. Previous studies have shown how surviving cancer has negative effects on labour market participation and employability. For example, compared to cancer-free controls of similar age, cancer survivors present reduced work ability, leading to higher unemployment rates^23^. Moreover, a recent study found that, among cancer survivors, low-educated males and workers employed in jobs requiring manual skills have the lowest probability of employment 4 years after diagnosis^24^. However, the future long term employment consequences and their shape over time hasn’t been addressed with life course approach methodologies while comparing the future working life after cancer with both workers with and without a disease.

Our hypothesis is that salaried workers who had cancer, recognised by a sickness absence (SA), are less likely to accumulate employment days in a stable trajectory when RTW than workers with SA due to other diagnoses or workers without SA. The objective of the present study was to evaluate differences in the probability of accumulating days of employment and employment participation trajectories (EPTs) in a sample of salaried workers in Catalonia (Spain) who had a SA due to cancer and compare them to workers who had a SA due to other diagnoses or no SA.

## Methods

We performed a register-based cohort study among 1,548 salaried workers living in Catalonia (675 men and 873 women) from the Spanish WORKss cohort^25^, which is part of the Continuous Working Life Sample (CWLS), an annual random representative sample of 4% of affiliates of the Spanish social security system. Data available from the CWLS enables reconstruction of working life since 2006 based on the known information, such as occupational category, economic activity, employment status, and employment conditions (i.e., employment, unemployment, type of contract, income, and working time), social benefits (i.e., unemployment, permanent disability, and retirement), other work-related variables (i.e., company ownership and size), and date of death. Moreover, the Catalan Institute for Medical and Health Evaluations provided information related to SA records, including the medical diagnosis of the episode coded according to the 10th edition of the International Classification of Diseases (ICD-10), as well as the starting and ending date^26^.

The final sample included salaried workers who had had a SA due to a malignant neoplasm (ICD-10, C00-C97) between 2012 and 2015 (N = 516, 225 men and 291 women). For each individual from the WORKss cohort, we selected two comparison workers from the same week of that the SA due to cancer ended (i.e., similar onset of time at risk) and also matched by age (within a 5-year range) and sex. First, a salaried worker with a SA due to a medical diagnosis different from cancer (ICD-10: A00-U99, except C00-C97; N = 516, 225 men and 291 women) was selected, and then another salaried worker without a SA at that moment (N = 516, 225 men and 291 women; Supplementary Table 1). The average age of each of the three comparison groups in 2012 was 49.8 for men (standard deviation: 9.96) and 47.0 for women (standard deviation: 9.44).

The study period covered from the date the workers entered the cohort until December 31, 2018. The length of the study period for salaried workers who RTW after SA ranged from 3 to 6 years considering complete years (the final part of the period was censored). Each worker was followed until they ended employment because they became unemployed, retired, were recognised as having a permanent disability, or died, or until the end of the follow-up. If workers had discontinued employment periods during the study, all of them were added. We also had information about working life since 2006 until the end of the follow-up period.

RTW was assessed by the accumulated days of employment during the follow-up period (entrance to 31/12/2018). Potential confounders considered in our analysis were the occupational category (non-manual skilled, non-manual non-skilled, manual skilled, or manual non-skilled); working time categorised as a percentage of weekly hours (full-time [> 87.5%], part-time [50–87.5%], or short and marginal part-time [≤ 37.5%-49%]); type of contract (permanent or temporary); monthly average income in tertiles (high [> 2370.0 €], medium [1450.0–2370.0 €], or low [≤ 1450.0 €]); company size (small/medium [up to 100 employees] and big [> 100 employees]); company ownership (private and public); and economic activity (primary sector [agriculture, hunting, forestry, fishing, mining, and quarrying]; manufacturing, and services). We also considered the previous 5-year employment ratio expressed as a percentage of employed days to the total potential working days, including working or unemployed or not affiliated days. Workers who changed categories over time were assigned the category in which they spent most of the follow-up period.

Patients were not involved in any stage of the study. Confidentiality was maintained in both databases. The authors received data that were previously anonymised.

### Statistical analysis

The sample was described according to response, explicative variables, and covariates mentioned above, and the chi-squared test was applied to assess significance between comparison groups. Negative binomial regression models were used to compare the probability of accumulating days of employment in salaried workers with SA due to cancer to workers with SA due to other diagnoses and without SA after testing for overdispersion through goodness-of-fit, which reports deviance, and Pearson chi-squared statistics. The estimator of this analysis, taking SA due to cancer as the reference group, was the incidence rate ratio (IRR), either the crude ratio or the ratio adjusted by all potential confounders mentioned above, and its 95% confidence interval (CI).

A second analysis was carried out to assess employment participation trajectories (EPTs) during follow-up in the three groups, applying latent class growth analysis (LCGA)^27^. The EPTs were obtained based on annually accumulated days of employment and estimated by assuming that they followed a quadratic function because it fit our data better than a linear function^28^. The optimal number of trajectories was chosen by considering the lowest Bayesian information criterion (BIC) and using the Lo-Mendell-Rubin adjusted and bootstrap likelihood ratio test (LMR-LRT)^29^. These tests indicated that the 4- and 3-trajectory model well-represented the EPT of our sample in both sexes. Nevertheless, due to the size of some of the trajectories and the principle of parsimony, we chose 3-trajectory models (Supplementary Table 2). The resulting EPT trajectories were described according to all potential confounders mentioned above, and chi-squared was applied to assess significance between EPTs. Finally, to measure the association between having a SA due to cancer and EPTs versus the comparison groups, we applied multinomial logistic regression with its relative risk ratio (RRR) and 95% CI.

All analyses were stratified by sex. Stata v.13 software was used for negative binomial models and multinomial regression models, and R version 4.1.0 and Mplus v.7 software were used for LCGA.

### Ethics approval and consent to participate

This study was performed in accordance with the standards of Good Clinical Practice and the principles of the Declaration of Helsinki. The study protocol guaranteed the fulfilment of Regulation (EU) 2016/679 of the European Parliament and the Council of 27 April 2016 on the protection of natural persons regarding the processing of personal data and the free movement of such data. It also fulfilled Spanish Organic Law 3/2018 of 5 December on the Protection of Personal Data and the Guarantee of Digital Rights. This study was approved by the Parc de Salut Mar Ethics Committee in Barcelona (Research Protocol no. 2020/9119) and exempted from informed consent requirements owing to its register-based design. The research team committed itself to the strict use of data for the present study. In addition, a linkage protocol agreement between the Centre for Research in Occupational Health at Pompeu Fabra University, the National Social Security Institute, and the Catalonian Institute for Medical Evaluations guaranteed the maintenance of confidentiality in providing the identified datasets to the authors.

## Results

Workers with SA due to cancer accumulated the fewest number of days of employment (3.2 years average for men and 3.7 years for women), whereas salaried workers without SA accumulated the highest number of days of employment (4.0 years average for men and 4.1 for women; Table 1). Salaried workers without SAs had a higher crude probability of continuing employment than those who had a SA due to cancer, especially among men (IRR 1.25, 95% CI 1.03‒1.52 vs. 1.10, 95% CI 0.97‒1.25 in women). A higher probability of continuing employment was also found among salaried workers who had a SA due to other diagnoses compared to those with SA due to cancer (men IRR 1.09, 95% CI 0.90‒1.32; women IRR 1.08, 95% CI 0.95‒1.23; Table 2). When IRR was adjusted individually, as well as by all potential confounders, the probability of employment remained higher in both men and women, but was only significant for men without SA.

**Table 1:** Employment-related characteristics among a sample of salaried workers with a SA due to cancer, SA due to other diagnoses, or no SA at all in Catalonia during the follow-up period (2012 and 2018), and previous employment 5 years prior to cohort entrance.

	Men (N = 675)				Women (N = 873)			
	SA cancer (N = 225)	SA other diagnoses (N = 225)	No SA any diagnoses (N = 225)		SA cancer (N = 291)	SA other diagnoses (N = 291)	No SA any diagnoses (N = 291)	
**Follow-up period**								
Total accumulated days of employment	262,869	286,245	328,939		393,825	425,556	434,249	

	N (%)	N (%)	N (%)	*p* value	N (%)	N (%)	N (%)	*p* value
**Contract type**								
Permanent	178 (79.1)	188 (83.6)	194 (86.2)	0.003**	243 (83.5)	233 (80.1)	243 (83.5)	0.206
Temporary	40 (17.8)	37 (16.4)	31 (13.8)		46 (15.8)	58 (19.9)	48 (16.5)	
**Working time (% weekly hours)**								
Full-time (> 87.5%)	184 (81.8)	195 (86.7)	190 (84.4)	0.037*	206 (70.8)	213 (73.2)	218 (74.9)	0.188
Part-time (50–87.5%)	12 (5.3)	13 (5.8)	11 (4.9)		59 (20.3)	48 (16.5)	45 (15.5)	
Short and marginal part-time (≤ 37.5–49%)	22 (9.8)	17 (7.6)	24 (10.7)		24 (8.2)	30 (10.3)	28 (9.6)	
**Monthly income average (tertiles)**								
High (> 2370.0 €)	105 (48.2)	90 (40.2)	91 (40.8)	0.364	79 (27.7)	70 (24.2)	74 (25.6)	0.360
Medium (1451.0–2370.0 €)	61 (28.0)	79 (35.3)	78 (35.0)		97 (34.0)	107 (37.0)	87 (30.1)	
Low (≤ 1450.0 €)	52 (23.9)	55 (24.6)	54 (24.2)		109 (38.3)	112 (38.8)	128 (44.3)	
**Occupational category**								
Non-manual skilled	67 (29.8)	38 (16.9)	51 (22.7)	< 0.0001***	94 (32.3)	53 (18.2)	68 (23.4)	< 0.0001***
Non-manual non-skilled	74 (32.9)	69 (30.7)	69 (30.7)		129 (44.3)	129 (44.3)	121 (41.6)	
Manual skilled	59 (26.2)	91 (40.4)	82 (36.4)		29 (10.0)	57 (19.6)	44 (15.1)	
Manual non-skilled	14 (6.2)	21 (9.3)	15 (6.7)		28 (9.6)	40 (13.8)	38 (13.1)	
**Economic activity**								
Agriculture, hunting, forestry, fishing, mining, and quarrying	1 (0.4)	*	4 (1.8)	0.002**	1 (0.3)	*	2 (0.7)	0.130
Manufacturing, energy construction	52 (23.1)	89 (39.6)	67 (29.8)		26 (8.9)	48 (16.5)	39 (13.4)	
Services	162 (72.0)	132 (58.7)	146 (64.9)		258 (88.7)	237 (81.4)	242 (83.2)	
**Company size**								
Small-medium (≤ 100 workers)	129 (57.3)	135 (60.0)	153 (68.0)	0.001**	158 (54.3)	143 (49.1)	170 (58.4)	0.058
Big (> 100 workers)	89 (39.6)	90 (40.0)	72 (32.0)		131 (45.0)	148 (50.9)	121 (41.6)	
**Company ownership**								
Private	161 (71.6)	179 (79.6)	175 (77.8)	0.001**	193 (66.3)	203 (69.8)	197 (67.7)	0.314
Public	44 (19.6)	29 (12.9)	28 (12.4)		64 (22.0)	57 (19.6)	53 (18.2)	
**5 years previous to follow-up**								
Employment time ratio (mean (SD))	90.9 (20.6)	93.1 (17.1)	93.2 (16.1)	0.019**	91.8 (17.7)	92.7 (17.2)	93.1 (16.1)	0.278

SA, sickness absence; Follow-up period ranged from 3 to 7 years from entrance to the cohort until end of 2018; Previous 5 years refers to each individual´s entrance; SD, standard deviation. *p < 0.05, **p < 0.01, ***p < 0.001.

**Table 2:** Probability of employment among salaried workers with a SA due to cancer (reference) and comparison groups adjusted individually by company characteristics and employment-related factors.

	Men			Women		
	IRR	95% CI	*p* value	IRR	95% CI	*p* value
**Crude**						
SA cancer	1			1		
SA other diagnoses	1.09	(0.90‒1.32)	0.386	1.08	(0.95‒1.23)	0.234
No SA any diagnoses	1.25	(1.03‒1.52)	0.022*	1.10	(0.97‒1.25)	0.134
**Individually adjusted by:**						
**Contract type**						
SA other diagnoses	1.06	(0.89‒1.28)	0.503	1.08	(0.95‒1.22)	0.226
No SA any diagnoses	1.21	(1.01‒1.46)	0.037*	1.09	(0.96‒1.24)	0.167
**Working time (% weekly hours)**						
SA other diagnoses	1.06	(0.88‒1.27)	0.564	1.08	(0.95‒1.22)	0.233
No SA any diagnoses	1.22	(1.02‒1.47)	0.032*	1.09	(0.97‒1.24)	0.161
**Income (tertiles)**						
SA other diagnoses	1.12	(0.94‒1.35)	0.203	1.07	(0.95‒1.20)	0.253
No SA any diagnoses	1.30	(1.09‒1.56)	0.004**	1.11	(0.99‒1.25)	0.071
**Occupational category**						
SA other diagnoses	1.05	(0.87‒1.27)	0.605	1.10	(0.97‒1.26)	0.134
No SA any diagnoses	1.20	(0.99‒1.45)	0.051	1.11	(0.98‒1.27)	0.101
**Economic activity**						
SA other diagnoses	1.06	(0.88‒1.28)	0.533	1.07	(0.94‒1.21)	0.297
No SA any diagnoses	1.25	(1.04‒1.50)	0.020*	1.09	(0.96‒1.24)	0.164
**Company size**						
SA other diagnoses	1.05	(0.88‒1.26)	0.594	1.07	(0.94‒1.21)	0.307
No SA any diagnoses	1.23	(1.02‒1.45)	0.026*	1.10	(0.97‒1.25)	0.141
**Company ownership**						
SA other diagnoses	1.05	(0.87‒1.27)	0.585	1.11	(0.97‒1.26)	0.134
No SA any diagnoses	1.23	(1.02‒1.48)	0.034*	1.09	(0.95‒1.24)	0.220
**Previous 5-year employment time (ratio)**						
SA other diagnoses	1.11	(0.91‒1.34)	0.299	1.07	(0.94‒1.21)	0.317
No SA any diagnoses	1.26	(1.04‒1.52)	0.018*	1.10	(0.97‒1.24)	0.155
**Adjustment by all variables**						
SA other diagnoses	1.09	(0.91‒1.30)	0.376	1.10	(0.97‒1.24)	0.130
No SA any diagnoses	1.27	(1.06‒1.53)	0.009**	1.09	(0.97‒1.23)	0.162

SA, sickness absence; IRR, incidence rate ratio; CI, confidence interval.

**Figure 1:**
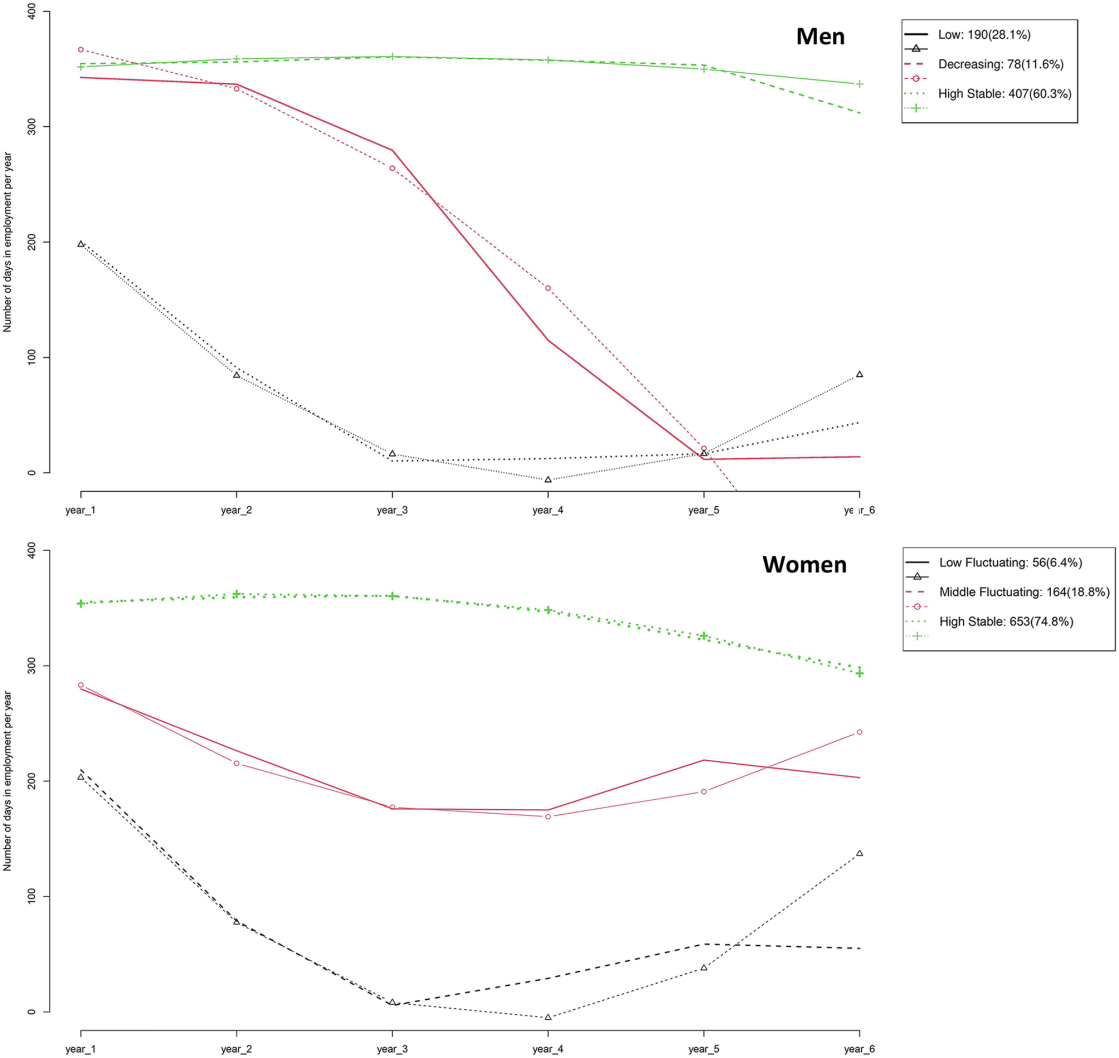
Observed and expected trajectories considering number of days in employment per year in a sample of salaried men (top) and women (bottom) in Catalonia (2012–2018).

When we assessed trends of the annual accumulation of days of employment, men and women exhibited different EPTs. Among men, the three EPTs were: Low (28.1% of workers), Decreasing (11.6%), and High Stable (60.3%; Fig. 1, Table 3). The Low EPT was characterised by the lowest accumulation of days of employment during the follow-up period, which coincided with the lowest 5-year previous employment (87.2%), the lowest ratio among trajectories and the highest proportion of temporary workers (26.2%), and working in small-medium companies (72.7%). The Decreasing trajectory represented the smallest group of people (11.6%), who tended to have a decreased number of annual accumulated days until reaching zero, with the highest proportion of short part-time arrangements (16.7%), manual non-skilled occupations (12.8%), and lowest income (28.2%). In contrast, the High Stable trajectory depicted high accumulation of days annually, with the best employment conditions among the three trajectories. In women, we identified three EPTs: Low Fluctuating (6.4% of workers), Middle Fluctuating (18.8%), and High Stable (74.8%; Fig. 1, Table 3). Unlike the trajectories found in men, women exhibited patterns of accumulation of days with steeper U shapes in both Low Fluctuating and Middle Fluctuating EPTs, which started with a higher accumulation of days that decreased steadily and then increased yearly. The Low Fluctuating trajectory was the least common EPT among women (6.4%) and showed the lowest accumulation of days of employment during the follow-up period, coinciding with the lowest 5-year previous employment (83.1%), the lowest proportion of high income (16.1%), and non-manual skilled workers (7.1%). The Middle Fluctuating trajectory represented older women, with the highest proportion of short and marginal part-time arrangements (17.9%) and the highest proportion of low-income workers (65.6%). Similar to the observation in men, among women, the High Stable trajectory was the most frequent EPT (74.8% of women) with the best employment conditions.

**Table 3:** Employment-related characteristics measured in the follow-up period (2012 and 2018) and previous employment measured 5 years before cohort entrance among a sample of salaried workers living in Catalonia across employment participation trajectories (EPTs) (2012–2018).

	Employment Trajectories							
	Men (N = 675)				Women (N = 873)			
	Low (28.1%)	Decreasing (11.6%)	High Stable (60.3%)	p value	Low Fluctuating (6.4%)	Middle Fluctuating (18.8%)	High Stable (74.8%)	*p* value
	N (%)	N (%)	N (%)		N (%)	N (%)	N (%)	
**Comparison groups**								
SA cancer	82 (36.4)	25 (11.1)	118 (52.4)		15 (5.2)	71 (24.4)	205 (70.5)	
SA other diagnoses	69 (30.7)	23 (10.2)	133 (59.1)		20 (6.9)	48 (16.5)	223 (76.6)	
No SA any diagnoses	39 (17.3)	30 (13.3)	156 (69.3)		21 (7.2)	45 (15.5)	225 (77.3)	
**Follow-up period**								
**Age in 2012 (years)**								
≤ 25	*	2 (2.6)	4 (10.0)	< 0.0001***	1 (1.8)	1 (0.6)	7 (1.1)	< 0.0001***
26–35	10 (5.3)	9 (11.5)	53 (13.0)		10 (17.9)	15 (9.2)	77 (11.8)	
36–45	19 (10.0)	9 (11.5)	92 (22.6)		21 (37.5)	29 (17.7)	232 (35.5)	
46–55	43 (22.6)	11 (14.1)	174 (42.8)		15 (26.8)	38 (23.2)	253 (38.8)	
> 55	118 (62.1)	47 (60.3)	84 (20.6)		9 (16.1)	81 (49.4)	84 (12.9)	
**Contract type**								
Permanent	135 (73.8)	59 (75.6)	366 (89.9)	< 0.0001***	40 (71.4)	111 (68.5)	568 (87.0)	< 0.0001***
Temporary	48 (26.2)	19 (24.4)	41 (10.1)		16 (28.6)	51 (31.5)	85 (13.0)	
**Working time (% weekly hours)**								
Full-time (> 87.5%)	146 (79.8)	61 (78.2)	362 (88.9)	0.013*	43 (76.8)	101 (62.4)	493 (75.5)	0.003**
Part-time (50–87.5%)	14 (7.7)	4 (5.1)	18 (4.4)		10 (17.9)	32 (19.8)	110 (16.9)	
Short and marginal part-time (≤ 37.5%-49%)	23 (12.6)	13 (16.7)	27 (6.6)		3 (5.4)	29 (17.9)	50 (7.65)	
**Monthly income average (tertiles)**								
High (> 2370.0 €)	57 (31.7)	22 (28.2)	207 (50.9)	< 0.0001***	9 (16.1)	29 (18.8)	185 (28.3)	< 0.0001***
Medium (1451.0—2370.0 €)	52 (28.9)	25 (32.1)	141 (34.6)		16 (28.6)	24 (15.6)	251 (38.4)	
Low (≤ 1450.0 €)	71 (39.4)	31 (39.7)	59 (14.5)		31 (55.4)	101 (65.6)	217 (33.2)	
**Occupational category**								
Non-manual skilled	42 (23.0)	14 (18.0)	100 (24.6)	0.199	4 (7.1)	35 (21.6)	176 (27.0)	0.001**
Non-manual non-skilled	59 (32.2)	23 (29.5)	130 (31.9)		31 (55.4)	60 (37.0)	288 (44.1)	
Manual skilled	57 (31.2)	28 (35.9)	147 (36.1)		13 (23.2)	26 (16.1)	91 (13.9)	
Manual non-skilled	18 (9.8)	10 (12.8)	22 (5.4)		4 (7.1)	32 (19.8)	70 (10.7)	
**Economic activity**								
Agriculture, hunting, forestry, fishing, mining, and quarrying	2 (1.1)	2 (2.6)	1 (0.3)	0.001**	1 (1.8)	1 (0.6)	1 (0.2)	0.013*
Manufacturing, energy construction	55 (29.0)	29 (37.2)	124 (30.5)		6 (10.7)	15 (9.2)	92 (14.1)	
Services	119 (62.6)	44 (56.4)	277 (68.1)		48 (85.7)	139 (84.8)	550 (84.2)	
**Company size**								
Small-medium (≤ 100 workers)	133 (72.7)	46 (59.0)	238 (58.5)	0.004**	30 (53.6)	102 (63.0)	339 (51.9)	0.041*
Big (> 100 workers)	50 (27.3)	32 (41.0)	169 (41.5)		26 (46.4)	60 (37.0)	314 (48.1)	
**Company ownership**								
Private	135 (73.8)	61 (78.2)	319 (78.4)	0.003**	42 (75.0)	118 (72.8)	433 (66.3)	0.006**
Public	22 (12.0)	11 (14.1)	68 (16.7)		6 (10.7)	19 (11.7)	149 (22.8)	

	Mean (SD)	Mean (SD)	Mean (SD)		Mean (SD)	Mean (SD)	Mean (SD)	
**5 years previous to follow-up**								
Employment time (ratio)	87.2 (24.4)	90.8 (18.1)	95.1 (13.4)	0.001**	83.1 (24.8)	86.0 (23.7)	95.0 (13.1)	< 0.0001***
Total	190	78	407		56	164	653	

SA, sickness absence; Follow-up period ranged from 3 to 7 years, from entrance to the cohort until end of 2018; Previous 5 years was calculated from each individual´s entrance; SD, standard deviation. *p < 0.05, **p < 0.01, ***p < 0.001.

In Table 4, we examine the probability of belonging to each EPT for individuals who had a SA due to other diagnoses or no SA compared to those who had a SA due to cancer. Among men, we found that individuals without a SA had 2.78 (95% CI 1.77‒4.36) times the probability of belonging to the High Stable EPT than the Low EPT compared to men who had a SA due to cancer, and 2.52 (95% CI 1.31‒4.85) times the probability of belonging to the Decreasing EPT than the Low EPT (Table 4). When adjusted for all potential confounders, the probability of men without a SA belonging to the High Stable EPT rather than the Low EPT was found 3.21 (95% CI 1.87‒5.50) times higher than for men who had a SA due to cancer. In contrast to men, women without a SA had less probability of belonging to a Middle Fluctuating EPT than to a Low Fluctuating EPT than women who had a SA due to cancer (RRR 0.45, 95% CI 0.24‒0.97). Adjusting for potential confounders did not vary the direction of the association. Workers with SA due to other diagnoses followed the same trend as workers without SAs but had lower estimates and weaker evidence, especially when adjusted for potential confounders.

**Table 4:** Probability of belonging to employment participation trajectories (EPTs) among salaried workers with a SA due to cancer (reference) and comparison groups.

	Men						Women					
	Decreasing vs. Low			High Stable vs. Low			Middle Fluctuating vs. Low Fluctuating			High Stable vs. Low Fluctuating		
	RRR	95% CI	*p* value	RRR	95% CI	*p* value	RRR	95% CI	*p* value	RRR	95% CI	*p* value
**Crude**												
SA cancer	1			1			1			1		
SA other diagnoses	1.09	(0.57‒2.10)	0.788	1.34	(0.89‒2.01)	0.157	0.51	(0.24‒1.09)	0.081	0.82	(0.41‒1.64)	0.566
No SA any diagnoses	2.52	(1.31‒4.85)	0.006**	2.78	(1.77‒4.36)	< 0.0001***	0.45	(0.21‒0.97)	0.041*	0.78	(0.39‒1.56)	0.489
**Individually adjusted by:**												
**Contract type**												
SA other diagnoses	1.00	(0.52‒1.92)	0.997	1.21	(0.80‒1.84)	0.374	0.51	(0.24‒1.11)	0.089	0.86	(0.43‒1.74)	0.682
No SA any diagnoses	2.30	(1.19‒4.44)	0.013	2.50	(1.56‒3.93)	< 0.0001***	0.46	(0.22‒0.99)	0.048*	0.80	(0.40‒1.60)	0.527
**Working time (% weekly hours)**												
SA other diagnoses	1.01	(0.52‒1.94)	0.976	1.21	(0.80‒1.83)	0.371	0.50	(0.25‒1.21)	0.081	0.81	(0.40‒1.62)	0.549
No SA any diagnoses	2.30	(1.19‒4.43)	0.013*	2.57	(1.63‒4.07)	< 0.0001***	0.46	(0.21‒0.98)	0.044*	0.78	(0.39‒1.55)	0.475
**Income (tertiles)**												
SA other diagnoses	1.00	(0.52‒1.93)	0.993	1.32	(0.86‒2.03)	0.206	0.53	(0.25‒1.15)	0.108	0.84	(0.42‒1.69)	0.625
No SA any diagnoses	2.41	(1.24‒4.67)	0.009**	2.96	(1.83‒4.78)	< 0.0001***	0.45	(0.21‒0.98)	0.043*	0.85	(0.42‒1.70)	0.643
**Occupational category**												
SA other diagnoses	0.94	(0.48‒1.82)	0.856	1.26	(0.83‒1.91)	0.286	0.52	(0.23‒1.16)	0.108	0.95	(0.46‒1.79)	0.888
No SA any diagnoses	2.23	(1.15‒4.33)	0.017*	2.60	(1.64‒4.11)	< 0.0001***	0.44	(0.20‒0.98)	0.043*	0.86	(0.42‒1.79)	0.695
**Economic activity**												
SA other diagnoses	0.90	(0.46‒1.76)	0.764	1.23	(0.81‒1.87)	0.331	0.50	(0.23‒1.08)	0.079	0.78	(0.39‒1.57)	0.491
No SA any diagnoses	2.33	(1.18‒4.61)	0.015*	3.01	(1.87‒4.85)	< 0.0001	0.49	(0.23‒1.06)	0.070	0.82	(0.41‒1.64)	0.570
**Company size**												
SA other diagnoses	1.01	(0.52‒1.95)	0.976	1.24	(0.82‒1.88)	0.314	0.53	(0.25‒1.14)	0.106	0.81	(0.41‒1.63)	0.561
No SA any diagnoses	2.50	(1.28‒4.84)	0.007**	2.75	(1.73‒4.37)	< 0.0001***	0.46	(0.21‒0.98)	0.045*	0.79	(0.39‒1.57)	0.495
**Company ownership**												
SA other diagnoses	0.98	(0.50‒1.92)	0.949	1.25	(0.81‒1.93)	0.317	0.43	(0.19‒0.98)	0.046	0.76	(0.35‒1.62)	0.472
No SA any diagnoses	2.15	(1.07‒4.35)	0.032*	2.82	(1.72‒4.63)	< 0.0001***	0.41	(0.18‒0.94)	0.034	0.73	(0.34‒1.56)	0.419
**Previous 5-year employment time (ratio)**												
SA other diagnoses	1.07	(0.56‒2.06)	0.834	1.29	(0.85‒1.94)	0.233	0.51	(0.24‒1.09)	0.081	0.80	(0.40‒1.63)	0.541
No SA any diagnoses	2.50	(1.29‒4.79)	0.006**	2.67	(1.69‒4.22)	< 0.0001***	0.44	(0.20‒0.94)	0.035	0.75	(0.37‒1.51)	0.421
**Adjustment by all variables:**												
SA other diagnoses	0.82	(0.41‒1.66)	0.583	1.09	(0.77‒2.00)	0.379	0.53	(0.22‒1.29)	0.164	0.97	(0.43‒2.18)	0.944
No SA any diagnoses	2.14	(1.04‒4.43)	0.040*	3.21	(1.87‒5.50)	< 0.0001***	0.46	(0.19‒1.11)	0.083	0.90	(0.40‒0.01)	0.794

SA, sickness absence; RRR, relative risk ratio; CI, confidence interval. *p < 0.05, **p < 0.01, ***p < 0.001.

## Discussion

This study shows that salaried workers with a SA due to cancer were less likely to accumulate employment days after ending the absence than those without a SA. This association exhibited a clearer positive pattern in men than in women. In women, we found weaker evidence with lower estimates than men. Similarly, both men and women with SA due to other diagnoses were more likely to be employed than workers with SA due to cancer. Furthermore, trajectories of employment showed that male salaried workers without a SA were 3-times more likely to be part of a stable employment trajectory than men with a SA due to cancer. These association patterns persisted after adjusting for company characteristics and employment-related factors.

Employability differences between cancer survivors and comparison groups were what we expected. The RTW population that has survived cancer have a lower probability of maintenance and accumulation of days of employment than the rest of the working population. These results did not substantially vary when adjusted for employment-related factors and company characteristics, which indicates that the lower employability is probably due to side effects provoked by cancer and its treatment (physical and psychological). Previous studies have shown that most cancer survivors RTW after treatment^30, 31^, but not whether they continued employed in the long-term after RTW. One prior review found that cancer survivors are more likely to be unemployed than the general working population^23^. However, the authors also found that the unemployment rate among cancer survivors was 34%, meaning that, even though it was higher than in the general working population, most of them continue in employment after a cancer diagnosis.

Men with SA due to other diagnoses present a gradient of future employability that is more favourable in terms of future employment accumulation than in workers with a SA due to cancer but worse compared to workers without SAs. Very few studies have addressed differences in work consequences among other diseases versus cancer. Nonetheless, there is a growing body of literature about chronic diseases as a whole, including cancer and RTW^32, 33^, which is much more action oriented (i.e., comparison of RTW interventions) than cancer research. Our results suggest differences between cancer and other chronic diseases. Furthermore, a qualitative study of employers’ perspectives of RTW among cancer survivors, found that this condition has a different status than others due to a lack of questioning the diagnosis, the immediate thought of the risk of death, and high psychological demands of the disease^34^. In this study, we considered all types of diagnoses in the comparison group regardless of the chronicity or duration of the SA to assess the overall effect of SA on future employment.

Studies carried out in northern European countries have found that younger age, higher levels of education, absence of surgery, fewer physical symptoms, shorter duration of sick leave, male gender, and Caucasian ethnicity are variables associated with RTW^21^, but their effect on employment in the long-term has not been studied. Regarding work-related factors, perceived employer accommodation has been found to be a strong predictor^30^.

EPTs revealed differences regarding employment trajectories after RTW. Most workers with a SA due to cancer, both men and women, go back to their jobs and stay employed, which is in agreement with previous literature^30^. The trajectory that represented the most stable labour life in our study (High Stable trajectory) included the highest proportion of young workers with a more stable working life in terms of the type of contract and employment before the follow-up period, which could explain the continuation in employment. Younger patients with cancer, even with side effects, may have remained in the labour market longer because they were too young to retire or to give up their professional career compared to older people. In addition, this stable labour life after cancer could represent less severe cancer, less aggressive treatments, and a better response in younger cancer patients. Other studies have found that younger age and locations associated with younger ages are factors associated with the likelihood of being employed and RTW^30^. We found higher levels of stable employment among women compared to men with SAs due to cancer, which is not what we expected. Previous studies have shown that female gender is a barrier to employment after cancer^30^. One explanation could be that some female cancer survivors may discontinue work because their partner is the main wage earner and provider of health insurance^35^, or can ensure the household’s financial stability alone^36^, so this results may vary by socioeconomic status. Existing literature shows discrepancies on the effect of survivors’ marital and relationship status, while some studies claim that marital status and gender don’t affect the probability of RTW^37, 38^, others find that marriage affects RTW positively in men. A recent study found that married women with breast cancer returned more to work than non-married ones, only among women over 50 years of age. In this same study, living with a partner showed decreasing trends of RTW and working time after RTW among women with equal household, highlighting the important role of financial support through higher freedom for a more flexible process of RTW^39^. Furthermore, there is also literature that has found higher odds of divorce when cancer diagnoses affected female comparing to men^40^, which could also be affecting women’s choice to go back and remain employed. Differences found with previous literature could be partly explained by age, as the women in our sample were younger, and as previously mentioned younger age is related to a greater employment likelihood. Previous research on the field has been carried out in countries where labour market dynamics and welfare state characteristics differ from the Spanish one, such as northern European countries. In fact, the gender gap in workforce participation is higher in Catalonia, as in the rest of Spain, than in these countries. In 2020, in Catalonia women employment rate was 63.3% and 70.9% in men^41^, contrasting Nordic countries such as Sweden with 78.3% in women and 83.2% in men^42^. This lower participation of women in employment could explain the differences with other studies since the ones that are on the labour market may have a higher engagement.

In men, we found a trajectory of reduced days of employment throughout the years until accumulating none (Decreasing trajectory). This trajectory represented the smallest proportion of the sample but comprised a group of manual workers. We think that they probably tried to return and maintain their jobs but cancer and its treatment affected their physical capacity, so they reduced their working time until they decided to exit the labour market. Previous studies have shown less probability of being employed in lower occupational categories due to high physical demands^43, 44^, and probably also due to more unstable employment trajectories. In women, this pattern exhibited a different evolution and depicted a more frequent future labour market trajectory characterised by women in a more precarious employment situation in terms of shortest working time and manual non-qualified occupations which could have made them reduce employment in the early years of cancer and increase it later, maybe because they needed the jobs in the first place.

We also identified a third trajectory comprising a group of men and women who accumulated the fewest number of days (Low and Low Fluctuating trajectories, respectively). In men, this sample stopped accumulating days of employment the third year, with a slight increase in the sixth year from the end of the SA due to cancer, probably because workers who were older preferred an exit from the labour market because working with side effects when they are just about to reach retirement age is not worthwhile. In this sense, a study carried out in the UK in men who survived prostate cancer found early retirement to be 9-times more likely in older men (aged 55–60 years) than men aged < 50 years^45^.

The main limitation of the present study is that the only information that we had regarding cancer is the SA diagnosis for the period 2012–2015. Therefore, we could not account for clinical features, such as type of treatment, stage of cancer, and the effects of prior health status regarding other comorbidities or cancer (first diagnosis, previous cancers, etc.) on the course of future employability. In addition, many individuals shifted between categories over the follow-up period and, in these cases, we assigned them to the category of explanatory and adjustment variables in which they spent most of their time during the follow-up. This could have led to a misclassification bias that underestimates the accumulation of SA days in other categories.

We also lacked information on workplace accommodations or support that have been proven to be determinant factors in employment sustainability and RTW^30^. Our sample size is small in relation to cancer cases certified by SAs, which could compromise the significance of our results. The self-employed were not included in the study, since by not being included in the general social security scheme, they are not entitled to sickness benefits.In addition, the methodology applied to employment trajectories involved group-based analyses that classified individuals according to similar characteristics. Consequently, some of the resulting groups had a very small number of observations, and these results should be interpreted with caution. However, some authors argue that a minimum of 5% should be enough to consider a pattern, and our results are above these recommendations^46^. The comparison groups resulted from matching show higher differences in men than in women regarding employment conditions and company characteristics. These differences shouldn’t be affecting our results since models are adjusted by them.

Our study also has numerous strengths. The study was conducted using a large administrative database, allowing us to select diagnosis subgroups and longitudinally study their trajectories with an extensive time window. Moreover, the size of the database allowed us to match our workers with SAs due to cancer to two comparison groups by age, sex, and follow-up time. This match allowed us to compare the working life of cancer survivors to the general working population with or without SAs, allowing us to account not only for the disease, but for the effect of SA. The diagnoses causing SAs were medically certified by primary doctors rather than self-reported, enhancing the validity of our results^47^.

To the best of our knowledge, employment continuation in a longitudinal sample of workers up to 6 years after the end of the SA has not previously been compared to the general salaried working population and workers who had a SA due to cancer for the same follow-up length and calendar days. More research is needed to understand the consequences of the disease and design interventions that address working difficulties caused by chronification of cancer in the long-term, not only at the beginning of the process of RTW.

## Conclusions

Focusing on RTW after cancer treatment as a binary decision ignores the complexity of relationships between health and work in the development of working life at a later stage of survivorship. Our study shows that workers with SA due to cancer continue working after SA, though in a lower proportion than matched controls and with a less stable employment trajectory. Thus, this study constitutes a step towards further understanding the relationship between cancer and employment in the long-term and encourages future research in this area.

## Supplementary Information

Supplementary Information.

## Data Availability

The data that support the findings of this study are available but restrictions apply to the availability of these data, which were used under license for the current study, and so are not publicly available. Administrative data come from an annual sample of Spanish Social Security affiliates that are part of the Continuous Working Life Sample (CWLS) provided by the General Directorate of Social Security (DGOSS) and linked records of sickness absence episodes certified in Catalonia provided by Catalan Institute for Medical and Health Evaluations (ICAM). The record linkage was possible thanks to an agreement signed between DGOSS, ICAM, and CiSAL-UPF, maintaining data confidentiality.
