# Supplementary material for: Returning to work after a sickness absence due to cancer: a cohort study of salaried workers in Catalonia (Spain)
Source: Sci Rep. 2021 Dec 14;11:23953. doi: 10.1038/s41598-021-03368-8 (PMC8671387; doi:10.1038/s41598-021-03368-8)
Supplement: Supplementary file 1 — Supplementary Information. [file 41598_2021_3368_MOESM1_ESM.docx]

**SUPPLEMENTARY MATERIAL:**

| Table 1: Cancer location in the group of workers with SA due to cancer and diagnosis underlying SAs in the comparison group with other causes by sex in Catalonia (2012-2015) | | | | | | | | | |  |
| --- | --- | --- | --- | --- | --- | --- | --- | --- | --- | --- |
|  |  |  |  |  |  |  |  |  |  |  |
| **Comparison group** | | | | | | | **Men** | | **Women** |  |
| **SA due to cancer diagnosis** | | | | | | | N (%) | | N (%) |  |
| Lip, oral cavity, and pharynx | | | | | | | 3 (1.3) | | 2 (0.7) |  |
| Digestive organs | | | | | | | 42 (18.7) | | 25 (8.6) |  |
| Respiratory system | | | | | | | 19 (8.4) | | 6 (2.1) |  |
| Bone and articular cartilage | | | | | | | 1 (0.4) | | * |  |
| Skin | | | | | | | 27 (12.0) | | 53 (18.2) |  |
| Connective and soft tissue | | | | | | | 3 (1.3) | | * |  |
| Breast and female genital organs | | | | | | | 1 (0.4) | | 152 (52.2) |  |
| Male genital organs | | | | | | | 52 (23.1) | | * |  |
| Urinary organs | | | | | | | 41 (18.2) | | 17 (5.8) |  |
| Eye, brain, and central nervous system | | | | | | | 10 (4.4) | | 5 (1.7) |  |
| Endocrine glands and related structures | | | | | | | 6 (2.7) | | 15 (5.2) |  |
| Secondary and ill-defined | | | | | | | 6 (2.7) | | 7 (2.4) |  |
| Lymphoid, haematopoietic, and related tissue | | | | | | | 14 (6.2) | | 8 (2.7) |  |
| Multiple locations | | | | | | | * | | 1 (0.3) |  |
| **Total** | | | | | | | 225 (100) | | 291 (100) |  |
| **SA due to other diagnoses** | | | | | | | N (%) | | N (%) |  |
| Infectious and parasitic diseases | | | | | | | 33 (14.7) | | 32 (11.0) |  |
| In situ, benign neoplasms | | | | | | | * | | 9 (3.1) |  |
| Endocrine, nutritional, and metabolic diseases | | | | | | | * | | 2 (0.7) |  |
| Mental and behavioural disorders | | | | | | | 15 (6.7) | | 22 (7.6) |  |
| Diseases of the nervous system | | | | | | | 2 (0.9) | | 12 (4.1) |  |
| Diseases of the eye and adnexa | | | | | | | 4 (1.8) | | 6 (2.1) |  |
| Diseases of the ear and mastoid process | | | | | | | 4 (1.8) | | 7 (2.4) |  |
| Diseases of the circulatory system | | | | | | | 10 (4.4) | | 12 (4.1) |  |
| Diseases of the respiratory system | | | | | | | 46 (20.4) | | 47 (16.2) |  |
| Diseases of the digestive system | | | | | | | 17 (7.6) | | 19 (6.5) |  |
| Diseases of the skin and subcutaneous tissue | | | | | | | 2 (0.9) | | 7 (2.4) |  |
| Diseases of the musculoskeletal system and connective tissue | | | | | | | 59 (26.2) | | 68 (23.4) |  |
| Diseases of the genitourinary system | | | | | | | 10 (4.4) | | 10 (3.4) |  |
| Pregnancy, childbirth, and the puerperium | | | | | | | 1 (0.4) | | 4 (1.4) |  |
| Symptoms, signs, and abnormal clinical and laboratory findings | | | | | | | 4 (1.8) | | 17 (5.8) |  |
| Injury, poisoning, and certain other consequences of external causes | | | | | | | 17 (7.6) | | 16 (5.5) |  |
| Factors influencing health status and contact with health services | | | | | | | 1 (0.4) | | 1 (0.3) |  |
| **Total** | | | | | | | 225 (100) | | 291 (100) |  |
| *N, Absences between 2012 and 2015; SA, sickness absence; MD(P25, P75), median duration and percentile 25 and 75 of the absences. | | | | | | | | | |  |
| Table 2: Goodness-of-fit indicator values for each model class | | | | | |  |  |  |  |  |
|  | **Model** | **E** | **BIC** | **LMR-LRT** | **% individuals in each class** |  |  |  |  |  |
| **Men** | 2-class | 0.992 | 37.225.198 | 0.0000*** | 69.7, 30.3 |  |  |  |  |  |
|  | **3-class** | **0.985** | **36.317.827** | **0.0340*** | **60.3, 11.6, 28.1** |  |  |  |  |  |
|  | 4-class | 0.981 | 35.642.475 | 0.0226* | 14.5, 14.2, 60.3, 11.0 |  |  |  |  |  |
| **Women** | 2-class | 0.997 | 46.623.942 | 0.0000*** | 77.3, 22.7 |  |  |  |  |  |
|  | **3-class** | **0.997** | **45.720.904** | **0.0075**** | **6.4, 18.8, 74.8** |  |  |  |  |  |
|  | 4-class | 0.970 | 45.334.979 | 0.0071** | 5.8, 6.6, 18.7, 68.8 |  |  |  |  |  |
| E, entropy; BIC, Bayesian information criterion; LMR-LRT, Lo-Mendell-Rubin adjusted likelihood ratio test. The preferred 3-class model is presented in bold.*p<0.05, **p<0.01, ***p<0.001. | | | | | |  |  |  |  |  |
|  |  |  |  |  |  |  | |  |  |  |
